# Myocardial Injury in Children with Unoperated Congenital Heart Diseases

**DOI:** 10.1155/2015/104818

**Published:** 2015-11-16

**Authors:** Mohamed O. Hafez, Saed M. Morsy, Ragab A. Mahfoz, Ahmed R. Ali

**Affiliations:** ^1^Pediatrics Department, Faculty of Medicine, Zagazig University, Zagazig 44519, Egypt; ^2^Cardiology Department, Faculty of Medicine, Zagazig University, Zagazig 44519, Egypt

## Abstract

*Background.* Children with congenital heart diseases (CHDs) may have a risk of developing myocardial injury caused by volume and pressure overload.* Objective.* To evaluate the incidence of myocardial injury in children with cyanotic and acyanotic CHDs using cTnI assay and to correlate it with different hemodynamic parameters.* Methods.* This study included 80 children with CHDs (40 acyanotic and 40 cyanotic) as well as 40 healthy children (control group). Serum cTnI levels were measured for patients and control. Pulmonary to systemic blood flow (Qp/Qs) and pulmonary to systemic arterial pressure (Pp/Ps) ratios were measured for children with CHDs during cardiac catheterization.* Results.* Sixty-four out of 80 patients with CHDs had myocardial injury as evidenced by increased cTnI. Serum cTnI was significantly higher in both cyanotic and acyanotic groups compared to control group (*p* < 0.05). Serum cTnI level significantly correlated with oxygen saturation (SpO_2_), ejection fraction (EF), Qp/Qs, and Pp/Ps ratios.* Conclusion.* The incidence of myocardial injury was high in children with CHDs. The use of cTnI for follow-up of children with CHDs may help early detection of myocardial injury and help early management of these cases.

## 1. Introduction

Congenital heart disease (CHD) represents a major health problem and the most common cause of congenital anomalies [1]. The estimated prevalence reaches up to 10 per 1000 live births [2–4].

In CHD, anatomical defects are associated with abnormalities in hemodynamic load and neurohormonal activation [5]. Few studies suggested that children with CHD may have significant myocardial cell injury. This injury may be due to acute pressure and volume overload or due to arterial desaturation [6].

Cardiac troponins are structural proteins regulating the relation between actin and myosin in skeletal muscle and cardiac myocytes. Troponins do not occur in extracellular space, so their presence in serum is a sensitive marker of cardiac myocytes injury [7]. Now, cardiac troponins are considered the gold standard markers for myocardial injury [8].

Few reports have described the role of cardiac troponins for evaluating hemodynamic load and myocardial injury in children with CHDs [9].

The aim of this study was to evaluate the incidence of myocardial injury in children with CHD, both cyanotic and acyanotic, using cardiac Troponin I (cTnI) assay and to correlate it with different hemodynamic parameters.

## 2. Subjects and Methods

This case-control study was carried out at pediatric and cardiology departments, during the period from April 2012 to April 2014. The study included 80 children with CHD, 40 with acyanotic CHD and 40 with cyanotic CHD. Forty healthy age and sex-matched children served as a control group.

Informed consent was taken from parents or care givers of children to be enrolled in the study. The study was approved by the Ethical Committee of Faculty of Medicine, Zagazig University, in compliance with the Declaration of Helsinki.

Children aged 1 month to 12 years with echocardiographic diagnosis of CHD were included in the study. Exclusion criteria included infants and children with any condition or disease state which may affect cardiac hemodynamics or functions other than CHDs including infective endocarditis, chronic renal or lung failure, children taking cardiotoxic drugs, and critically ill children.

All subjects included in this study were subjected to full history taking, detailed clinical examination, electrocardiography, plain chest radiography, echocardiography, and measurement of serum cTnI level. Cardiac catheterization was done for patients with CHDs.

Echocardiographic examination was performed according to the American Society of Echocardiography recommendations [10]. Cardiac catheterization was done under general anesthesia using a biplane Philips catheterization laboratory at cardiac catheterization unit. The ratio of pulmonary to systemic blood flow (Qp/Qs) and the ratio of pulmonary to systemic arterial pressure (Pp/Ps) were measured for children with CHD as well as measurement of pressure and oxygen saturation in all cardiac chambers.

Serum cTnI level was measured using enzyme linked fluorescent assay. Values below 0.01 ng/mL could not be measured quantitatively and were considered negative and expressed as 0.00 ng/mL.

Data were analyzed using Statistical Package for Social Sciences (SPSS) release 16. Nonparametric values were expressed as median and range and the medians of two groups were tested by Mann-Whitney *U* test. Qualitative data are expressed as number and percentage and chi-square test was used for testing association of qualitative data. Correlations were performed using Spearman's rank correlation. In all analyses, *p* < 0.05 was considered statistically significant.

## 3. Results

Table 1 shows demographic criteria and diagnosis of study population. There was no difference between studied groups as regards age and gender.

Hemodynamic data showed that VSD and PDA patients had the greater Qp/Qs while TGA, VSD and PDA patients had the greater Pp/Ps values (Table 2).

Myocardial injury, as evidenced by increased cTnI, was found in 64 out of 80 (80%) children with CHD. Myocardial injury occurred more frequently in acyanotic patients than cyanotic patients (82.5% versus 77.5%) with no significant difference (Table 3).

The median cTnI level was significantly higher in both acyanotic and cyanotic groups compared to control group. However, no difference was found between children with cyanotic and acyanotic CHD (Table 4).

**Figure 1:**
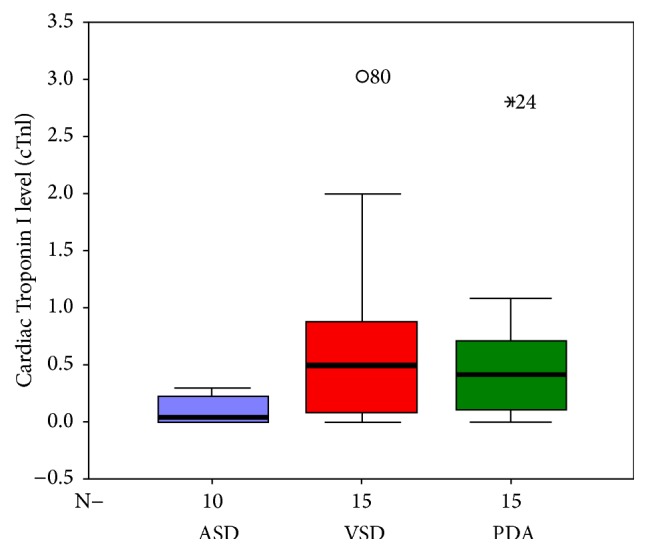
Box-plot shows cTnI levels in the ASD, VSD, and PDA patients. The bars represent the median and the 5th, 25th, 75th, and 95th percentiles.

**Figure 2:**
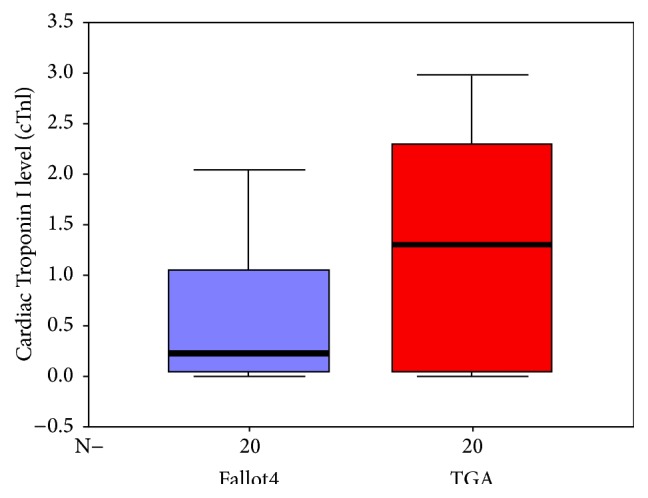
Box-plot shows cTnI levels in the TOF and TGA patients. The bars represent the median and the 5th, 25th, 75th, and 95th percentiles.

Regarding specific cardiac lesions, cTnI level was higher in patients with VSD and those with PDA compared to patients with ASD. However, no difference was found between patients with VSD and those with PDA (Table 4 and Figure 1). Also, serum cTnI level was significantly higher in patients with TGA than those with TOF (Table 4 and Figure 2).

Univariate analysis revealed significant positive correlations between cTnI levels and Qp/Qs and Pp/Ps ratio, whereas significant negative correlations were found between cTnI and SpO_2_ and EF (Table 5). No significant correlation was found between age and cTnI level.

## 4. Discussion

The current study demonstrated a high prevalence of myocardial injury in children with CHD reaching up to 80% using cTnI as a biomarker for this injury. The prevalence was 82.5% in acyanotic group and 77.5% in cyanotic group with no significant difference between both groups.

Uner et al. conducted a study to evaluate cTnI, N-terminal prohormone brain-type natriuretic peptide, and C-reactive protein levels in children with CHD. They found that prevalence of myocardial injury in children with CHD was 48.5%. It was found in 52% of patients with acyanotic CHD and in 37.5% of patients with cyanotic CHD [11].

Plausible explanation for this high incidence of elevated cTnI level in the current study is that, in Egypt and other developing countries, surgical correction of CHD is delayed due to limited resources. This delay leads to prolonged exposure of cardiac muscle to pressure and volume overloads giving rise to a high incidence of myocardial affection.

As regards cTnI levels in the current study, it was significantly higher in both cyanotic and acyanotic groups compared with the control group (*p* < 0.001).

Sugimoto et al. conducted their study to evaluate cTnI level in children with CHD and left-to-right shunt. The study showed an increased serum cTnI level in children with CHD [12].

Uner et al. found that cTnI was significantly higher in both cyanotic and acyanotic groups compared with the control group [11].

Plausible explanations for this injury include reduction of myocardial perfusion due to ventricular hypertrophy and myocardial extension [13]. Also, ventricular hypertrophy increases the oxygen demand of the myocardium, resulting in relative hypoperfusion of the myocardium [14].

Compared to acyanotic patients, the myocardium of cyanotic patients is exposed to more hypoxia, so higher cTnI levels were expected in cyanotic patients compared to acyanotic patients [11]. Nassef et al. found a significant difference in cTnI level between cyanotic and noncyanotic groups with cTnI level more in cyanotic group than acyanotic group [15].

In our study, cTnI level was higher in cyanotic group when compared to noncyanotic patients but it was nonsignificant statistically. Similar results were reported by Uner et al. [11].

This absence of significant difference between both groups as regards cTnI level in our study might indicate that the volume and pressure overload effects on cardiac muscle are equal to the effects of hypoxia regarding myocardial injury.

In the current study, cTnI level was significantly higher in VSD compared to ASD cases. Also, cTnI was significantly higher in PDA than ASD cases, but nonsignificant difference was found between VSD and PDA regarding cTnI level. Similar results were reported by Sugimoto et al. [12].

In the current study, the hemodynamic data showed that VSD and PDA patients had greater Qp/Qs and Pp/Ps than those of ASD patients. It means that VSD and PDA patients in this study were subjected to higher volume and pressure overload than those in ASD patients and it explains this difference in cTnI levels among these patients.

In contrast to these results, Uner et al. found nonsignificant difference between ASD and VSD patients as regards cTnI level [11]. Also, Tarkowska and Furmaga-Jabłońska found that cTnI levels in newborns with CHD did not depend on the type of the cardiac defects [16].

In our study, patients with TGA had significantly higher levels of cTnI than patients with TOF. This may be attributed to the high volume overload in patients with TGA.

Univariate analysis revealed significant positive correlation between cTnI levels and Qp/Qs and Pp/Ps ratio, whereas significant negative correlation was found between cTnI and oxygen saturation and EF. These results agree with those of Sugimoto et al. who found significant positive correlation between cTnI level and Pp/Ps ratio [12].

In contrast to these results, Eerolaa et al. found no correlation between cTnI level and oxygen saturation in children with hypoplastic left heart syndrome [17].

It is logic to assume that the patient with a longer period with volume or pressure load is more prone to develop myocardial injury. However, in this study there was no relation between age of the patients and cTnI levels; that is because the cases that presented late were the less severe ones (mostly ASD patients).

## 5. Conclusion

Our study showed a high incidence of myocardial injury in children with CHDs. The use of cTnI for follow-up of children with unoperated CHDs may help early detection of myocardial injury and help early management of these cases.

## Figures and Tables

**Table 1:** Demographic and clinical criteria of study population.

	Acyanotic group (*n* = 40)	Cyanotic group (*n* = 40)	Control group (*n* = 40)	*p*
Age (years), median (range)	5 (1.5–12)	4.5 (0.75–14)	4.25 (0.5–13)	NS^†^
Gender (male/female)	17/23	21/19	22/18	NS^‡^
Diagnostic categories, *n* (%)				
VSD	15 (37.5%)	—	—	—
ASD	10 (25%)	—	—	—
PDA	15 (37.5%)	—	—	—
TGA	—	20 (50%)	—	—
TOF	—	20 (50%)	—	—

VSD: ventricular septal defect; ASD: atrial septal defect; PDA: patent ductus arteriosus; TGA: transposition of the great arteries; TOF: Tetralogy of Fallot; NA: not available; †: Mann-Whitney *U* test; ‡: chi-square.

**Table 2:** Median of Qp/Qs and Pp/Ps in different cardiac lesions.

Cardiac defect	Qp/Qs median (range)	Pp/Ps median (range)
ASD	1.5 *(1.1–2.9)*	0.40 *(0.27–0.42)*
VSD	2.36 *(1.5–4.2)*	0.70 *(0.60–0.90)*
PDA	2.2 (1.4–2.96)	0.72 (0.43–0.75)
Fallot4	0.77 (0.62–1.1)	0.28 *(0.20–0.39)*
TGA	1.5 *(1.1–2.0)*	0.77 (0.2–0.92)

Qp/Qs: pulmonary to systemic blood flow; Pp/Ps: pulmonary to systemic arterial pressure; VSD: ventricular septal defect; ASD: atrial septal defect; PDA: patent ductus arteriosus; TGA: transposition of the great arteries; TOF: Tetralogy of Fallot.

**Table 3:** Incidence of increased cTnI level in study population.

	Increased cTnI level		*p*
	Present	Absent	
Study population, *n* (%)			
Patients (*n* = 80)	64 (80%)	16 (20%)	<0.001
Controls (*n* = 40)	0 (0%)	40 (100%)	
Patients, *n* (%)			
Acyanotic CHD (*n* = 40)	33 (82.5%)	7 (17.5%)	NS
Cyanotic CHD (*n* = 40)	31 (77.5%)	9 (22.5%)	

CHD: congenital heart diseases.

**Table 4:** Cardiac Troponin I levels in study population.

	Cardiac Troponin I level (ng/mL) median (range)	*p*
Study population		
Acyanotic CHD	0.41 (0–3.03)	*p*1 < 0.05
Cyanotic CHD	0.72 (0–3.0)	*p*2 < 0.001
Controls	0.00 (0.00–0.00)	*p*3 > 0.05
Acyanotic CHD		
VSD	0.50 (0.00–3.03)	*p*1^†^ < 0.001
PDA	0.42 (0.00–2.80)	*p*2^†^ < 0.001
ASD	0.04 (0.00–0.30)	*p*3^†^ > 0.05
Cyanotic CHD		
TOF	0.23 (0.00–2.00)	*p* ^‡^ < 0.05
TGA	1.30 (0.00–3.00)	

*p*1: acyanotic versus controls; *p*2: cyanotic versus controls; *p*3: acyanotic versus cyanotic patients; *p*1^†^: VSD versus ASD patients; *p*2^†^: PDA versus ASD patients; *p*3^†^: VSD versus PDA patients; *p* ^‡^: TOF versus TGA patients; CHD: congenital heart diseases; VSD: ventricular septal defect; ASD: atrial septal defect; PDA: patent ductus arteriosus; TGA: transposition of the great arteries; TOF: Tetralogy of Fallot.

**Table 5:** Univariate analysis of risk factors of myocardial injury in patients.

	*r*	*p*
Age (years)	−0.066	NS
SpO_2_ (%)	−0.375	<0.05
EF (%)	−0.378	<0.05
FS (%)	−0.147	NS
Qp/Qs	+0.445	<0.05
Pp/Ps	+0.496	<0.05

SpO_2_: oxygen saturation; EF: ejection fraction; FS: fractional shortening; Qp/Qs: pulmonary to systemic flow ratio; Pp/Ps: pulmonary to systemic peak pressure ratio.
